# Marked improvement of anti-N-methyl-D-aspartate receptor encephalitis by large-dose methylprednisolone and plasmapheresis therapy combined with ^18^F-fluorodeoxyglucose positron emission tomography imaging: A case report

**DOI:** 10.3892/etm.2014.1849

**Published:** 2014-07-16

**Authors:** BO CHEN, YIQI WANG, YU GENG, YUEHONG HUANG, SHUNYUAN GUO, XIAWA MAO

**Affiliations:** 1 Department of Neurology, Zhejiang Provincial People’s Hospital, Hangzhou, Zhejiang 310014, P.R. China; 2 Department of Urology, Zhejiang Provincial People’s Hospital, Hangzhou, Zhejiang 310014, P.R. China

**Keywords:** anti-N-methyl-D-aspartate receptor encephalitis, plasmapheresis, ^18^F-fluorodeoxyglucose positron emission tomography

## Abstract

Anti-N-methyl-D-aspartate receptor (anti-NMDAR) encephalitis, a recently defined and frequently misdiagnosed disease characterized by psychiatric symptoms, seizures, movement disorders and autonomic dysfunction, has been observed predominantly in young females with ovarian teratoma. Conventional imaging techniques, including computed tomography (CT) and magnetic resonance imaging (MRI), are often ineffective for diagnosis of the disease. If diagnosed early, the initiation of immunotherapy and removal of the tumor (if present) may result in recovery. The current study presents the case of a 38-year-old female with classic clinical symptoms of anti-NMDAR encephalitis. The MRI brain scan results were unremarkable, cerebral spinal fluid (CSF) biochemistry indicated non-specific lymphocytic pleocytosis and the CSF microbiology studies were negative. ^18^F-fluorodeoxyglucose positron emission tomography (^18^F-FDG PET) imaging revealed significant generalized asymmetric hypometabolism. The patient demonstrated marked recovery following treatment with a high dose of corticosteroids and plasmapheresis. Accordingly, the follow-up ^18^F-FDG PET imaging revealed significant improvement.

## Introduction

Anti-N-methyl-D-aspartate receptor (anti-NMDAR) encephalitis is a newly recognized autoimmune disorder that was first reported as paraneoplastic limbic encephalitis in 2007 (1). It is especially prevalent in young women with ovarian terotomas (2). However, more recent studies have revealed that it is not necessarily a paraneoplastic syndrome and may occur in patients without tumors (3). The clinical presentation of anti-NMDAR encephalitis includes acute psychiatric symptoms, cognitive disturbance, new onset of seizures, memory deficits, dyskinesia, dystonia, rigidity, ataxia and dysautonomia (4). Precise diagnosis may be established if anti-NMDAR antibodies are detected in the cerebral spinal fluid (CSF) and blood serum. There are no positive diagnostic features on brain magnetic resonance imaging (MRI) scans (5). Until recently, few studies have investigated the application of ^18^F-fluorodeoxyglucose positron emission tomography (^18^F-FDG PET) in the treatment of anti-NMDAR encephalitis. The present study describes a case of anti-NMDAR encephalitis that was diagnosed in a young female who presented subacute encephalitis and psychiatric manifestation without evidence of malignancy. The study was approved by the Ethics Committee of Zhejiang Provincial People’s Hospital (Hangzhou, China) and informed consent was provided by the patient.

## Case report

A 38-year-old female with no significant past medical history, was admitted to Zhejiang Provincial People’s Hospital due to the sudden onset of behavioral disturbance, speech problems and psychiatric symptoms one week previously. The patient was unable to answer simple questions and responded to all questions with meaningless answers. The patient also presented intermittent confusion associated with anxiety and agitation. The patient denied any problems with her eating and sleeping patterns, and reported no occurrence of hallucinations or seizures.

On examination, the patient was afebrile with normal vital signs. However, the patient was unable to answer simple questions and this was associated with intermittent confusion and agitation. There was no other evidence of any focal or global neurological deficit. The results of the patient analyses, including an MRI brain scan and a routine electroencephalography (EEG), were all normal. A lumbar puncture was performed to determine CSF biochemistry, cytology and microbiology. The CSF revealed an elevated white blood cell count of 25 cells/mm^3^. The CSF pressure, and the levels of protein and glucose were within normal range; the bacterial culture was negative. The patient was hospitalized, empiric treatment for viral encephalitis was initiated and acyclovir was administered intravenously.

A week following admission, the patient’s symptoms deteriorated. The patient was confused and became agitated more frequently; aggressive behavior towards family members also developed. The interaction with the surroundings was greatly reduced and occasionally the patient remained mute. There were signs of catatonia, including whole body rigidity and psychological pillow. Insomnia was apparent and the period spent sleeping decreased to 4 h/day. The patient also presented disturbances of the movement system, which included intermittent, involuntary movements of the facial muscles and tongue, and choreiform movements of the extremities. Lip smacking, lip pursing, chewing, sucking and teeth grinding was demonstrated. Fine muscle twitches of the right upper extremity were also observed as well as the paroxysmal upward gazing of the eyes without evident convulsion or jerking. The patient lost control of bladder and bowel movement and autoimmune encephalitis was highly suspected at this stage.

**Figure 1:**
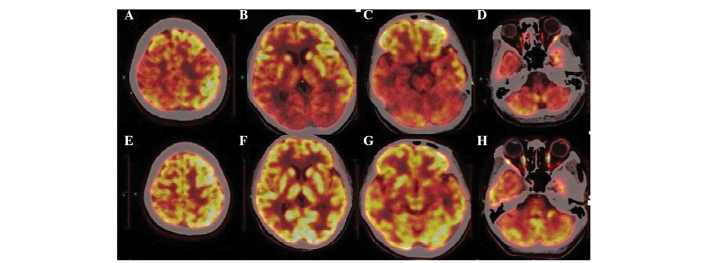
Selected images of the ^18^F-FDG PET brain scans. (A–D) Prior to treatment with high-dose corticosteroids and plasmapheresis, the scans revealed generalized hypometabolism without significant hypermetabolic foci. A significant asymmetrical reduction in the metabolism between the left and right hemispheres was noted, particularly in the temporal and parietal cortex. (E–H) The metabolic activities of the former areas were normalized following treatment, with the exception of a slightly lower metabolism in the right temporal cortex when compared with the contralateral site.

Repeated brain MRI scans enhanced with gadolinium, fluid-attenuated inversion recovery (FLAIR) and diffusion-weighted imaging (DWI) all appeared normal. The CSF was analyzed again; however, CSF biochemistry, cytology and microbiology were all within normal range. A CSF and serum anti-NMDAR antibody study was requested. Chest X-ray, computed tomography (CT) and abdominal ultrasound scans were unremarkable. The ^18^F-FDG PET whole body scan revealed no tumorous focus. However, the ^18^F-FDG PET brain scan demonstrated hypometabolism in the bilateral occipital cortex, parietal cortex, thalamus, right temporal cortex and left cerebellum (Fig. 1). A routine EEG taken immediately prior to the ^18^F-FDG PET scan revealed generalized slow waves with asymmetry in frequency and amplitude. Treatment with a large dose of intravenously administered methylprednisolone (1,000 mg/day) was initiated. However, the patient did not demonstrate any improvement within five days of treatment and the results of the serum and CSF anti-NMDAR antibody experiments returned positive. Plasmapheresis was subsequently attempted and there was marked improvement in the patient. The patient regained control of bladder and bowel movements following the first plasmapheresis treatment. Following three treatments with plasmapheresis, the patient was well oriented and able to respond to simple questions with a Mini Mental State Examination (MMSE) score of 10. Following five courses of plasmapheresis, the patient demonstrated significant recovery with a MMSE score of 24. A follow-up ^18^F-FDG PET scan revealed that glucose metabolism had returned to normal (Fig. 1). Routine EEG immediately prior to the ^18^F-FDG PET scan also demonstrated great improvement; α-like activity reappeared in the occipital area and the asymmetry of frequency and amplitude, as observed in previous EEG tests, had disappeared.

## Discussion

Anti-NMDAR encephalitis first was identified in 2007 by Dalmau *et al* (1), as treatment-responsive paraneoplastic encephalitis. Since then, >600 cases of anti-NMDAR encephalitis have been reported. Due to the acute or subacute onset of the disease, overlap of the stages and clinical symptoms and signs are extremely common. CSF biochemistry, cytology and microbiology examinations are either nonspecific or unremarkable. The MRI brain scan is unremarkable in 50% of cases, with the remainder demonstrating non-specific changes. EEG may reveal non-specific slowing, disorganized activity or epileptiform discharge. The diagnosis of anti-NMDAR encephalitis is highly reliant on clinical awareness and anti-NMDAR antibody detection in the CSF and serum.

Although the number of published studies on anti-NMDAR encephalitis has increased markedly since its discovery, only a few more recent studies have described the application of ^18^F-FDG PET in the evaluation of anti-NMDAR encephalitis (6–10). Leypoldt *et al* (6) revealed a characteristic change in cerebral glucose metabolism during NMDAR-antibody encephalitis of an increased frontotemporal-to-occipital gradient; however, no specific pattern of FDG PET imaging could be identified in the majority of cases (7–10). The majority of cases demonstrated various degrees of hyper- or hypometabolism of a particular area of the brain, which was associated with different stages of the disease and the different structures involved at each stage. Certain cases that underwent follow-up FDG PET imaging following treatment revealed an association between FDG PET imaging and clinical recovery of the patient. Greiner *et al* (7) reported the case of an 11-year-old female with a novel onset of explosive epilepsy. ^18^F-FDG imaging at 24 days following the onset of seizure revealed asymmetric hypermetabolism in the superior right frontal lobe. Maqbool *et al* (8) described a case of anti-NMDAR encephalitis where on day 26 following admission, ^18^F-FDG PET imaging demonstrated global hypometabolism and the presence of a prominent focally intense hypermetabolic lesion in the right cerebellar cortex. Clinical signs of improvement were observed following two courses of intravenous immunoglobulin therapy. A repeat brain FDG-PET scan on day 46 revealed an overall improvement, with focal hypometabolism in the right cerebellar cortex. Similar results were observed in the study by Pillai *et al* in 2010 (9). ^18^F-FDG PET images of two cases, which were taken in the sixth week and fifth month, respectively, following the onset of disease, demonstrated diffuse cerebral hypometabolism, including in the bilateral frontal, parietal, temporal and occipital lobe and thalamus areas.

In the present study, the ^18^F-FDG PET images of the patient revealed a general reduction in metabolism in the bilateral frontal, temporal and occipital lobes, thalamus and left cerebellum, without an apparent zone of hypermetabolism. The decreasing levels of metabolism in the left and right hemisphere were asymmetrical, particularly in the temporal and parietal cortex. An EEG study carried out immediately prior to the ^18^F-FDG PET imaging also revealed generalized Δ-θ slowing with significant frequency and amplitude asymmetry. Following successful treatment with a high dose of corticosteroids and subsequently, with five courses of therapeutic plasmapheresis over two weeks, the current patient improved significantly. A follow-up ^18^F-FDG PET image in the seventh week indicated that the metabolic activity of the cerebral and cerebellum had returned to normal. Furthermore a follow-up EEG also revealed great improvement. According to cases analyzed in previous studies and the case reported in the current study (6,8), the present authors hypothesize that the changes in the metabolism of the cerebrum and cerebellum are reversible in anti-NMDAR encephalitis. Furthermore, if MRI scans are negative, ^18^F-FDG PET imaging offers maximal efficacy for early diagnosis and the evaluation of prognosis for anti-NMDAR encephalitis.

Although no standard of treatment exists for anti-NMDAR encephalitis, eradication of the associated malignancy and immunotherapy is recommended. The immunotherapies include corticosteroids, intravenous immunoglobulin (IVIG), plasmapheresis, rituximab, cyclophosphamide and azathioprine, with corticosteroids, IVIG or plasmapheresis constituting the primary approach (11,12). However, systematic comparisons between the three first-line modalities are not yet available (12). If the primary therapies are ineffective, rituximab or cyclophosphamide may be considered as the second-line therapies. For patients without a malignancy, first-line therapy may not be effective. Therefore, second-line immunotherapy is usually required. In the present case study, the patient achieved considerable improvements following plasmapheresis initiated in the fourth week, following no effect from steroid treatment. This was consistent with a study by Pham *et al* (13) in which nine cases of anti-NMDA-R encephalitis were reviewed and two acquired the best recovery by undergoing plasmapheresis treatment. Thus, the authors of the current study continue to postulate that early plasmapheresis treatment may lead to beneficial treatment results.

In conclusion, ^18^F-FDG PET imaging is greatly advantageous in the treatment of anti-NMDAR encephalitis. The current study presented a special change in PET images during the overall process of the therapy, which has not been observed in previous case studies. However, it should be noted that the present study only analyzed one patient who was treated using plasmapheresis in combination with ^18^F-FDG PET. Further clinical investigations and studies of PET application in the treatment of anti-NMDAR encephalitis are required to confirm the results. Furthermore, when the therapeutic effect of steroid treatment is poor, early plasmapheresis helps to improve the prognosis of anti-NMDAR encephalitis.

## References

[1] Dalmau J, Tüzün E, Wu HY (2007). Paraneoplastic anti-N-methyl-D-aspartate receptor encephalitis associated with ovarian teratoma. Ann Neurol.

[2] Graus F, Dalmau J (2007). Paraneoplastic neurological syndromes: diagnosis and treatment. Curr Opin Neurol.

[3] Irani SR, Bera K, Waters P (2010). N-methyl-D-aspartate antibody encephalitis: temporal progression of clinical and paraclinical observations in a predominantly non-paraneoplastic disorder of both sexes. Brain.

[4] Vincent A, Bien CG (2008). Anti-NMDA-receptor encephalitis: a cause of psychiatric, seizure, and movement disorders in young adults. Lancet Neurol.

[5] Florance NR, Davis RL, Lam C (2009). Anti-N-methyl-D-aspartate receptor (NMDAR) encephalitis in children and adolescents. Ann Neuro1.

[6] Leypoldt F, Buchert R, Kleiter I (2012). Fluorodeoxyglucose positron emission tomography in anti-N-methyl-D-aspartate receptor encephalitis: distinct pattern of disease. J Neurol Neurosurg Psychiatry.

[7] Greiner H, Leach JL, Lee KH, Krueger DA (2011). Anti-NMDA receptor encephalitis presenting with imaging findings and clinical features mimicking Rasmussen syndrome. Seizure.

[8] Maqbool M, Oleske DA, Huq AH, Salman BA, Khodabakhsh K, Chugani HT (2011). Novel FDG-PET findings in anti-NMDA receptor encephalitis: a case based report. J Child Neurol.

[9] Pillai SC, Gill D, Webster R, Howman-Giles R, Dale RC (2010). Cortical hypometabolism demonstrated by PET in relapsing NMDA receptor encephalitis. Pediatr Neurol.

[10] Baumgartner A, Rauer S, Mader I, Meyer PT (2013). Cerebral FDG-PET and MRI findings in autoimmune limbic encephalitis: correlation with autoantibody types. J Neurol.

[11] Dalmau J, Lancaster E, Martinez-Hernandez E, Rosenfeld MR, Balice-Gordon R (2011). Clinical experience and laboratory investigations in patients with anti-NMDAR encephalitis. Lancet Neuro1.

[12] Titulaer MJ, McCracken L, Gabilondo I (2013). Treatment and prognostic factors for long-term outcome in patients with anti-NMDA receptor encephalitis: an observational cohort study. Lancet Neurol.

[13] Pham HP, Daniel-Johnson JA, Stotler BA, Stephens H, Schwartz J (2011). Therapeutic plasma exchange for the treatment of anti-NMDA receptor encephalitis. J Clin Apher.

